# PKIDB-informed molecular profiling improves reproducible prediction of cancer kinase-inhibitor response

**DOI:** 10.3389/fphar.2026.1923164

**Published:** 2026-09-17

**Authors:** Miao Liu, Jiayang Song, Xianbin Liu, Linlin Zhao, Yu Xiong

**Affiliations:** 1 Department of Clinical Laboratory, Mianyang Central Hospital, School of Medicine, University of Electronic Science and Technology of China, Mianyang, Sichuan, China; 2 NHC Key Laboratory of Nuclear Technology Medical Transformation (Mianyang Central Hospital), School of Medicine, University of Electronic Science and Technology of China, Mianyang, Sichuan, China; 3 Department of Pharmacy, Cangxi People’s Hospital, Guangyuan, Sichuan, China; 4 Department of Pharmacy, Mianyang Central Hospital, School of Medicine, University of Electronic Science and Technology of China, Mianyang, Sichuan, China; 5 The First Teaching Hospital of Tianjin University of Traditional Chinese Medicine, National Clinical Research Center for Chinese Medicine Acupuncture and Moxibustion, Tianjin, China; 6 Sichuan Provincial Engineering Research Center of Nuclear Medical Equipment Translation and Application, Mianyang Central Hospital, Mianyang, Sichuan, China

**Keywords:** DepMap, drug response prediction, external validation, GDSC, kinase inhibitor, pharmacogenomics, PKIDB, PRISM

## Abstract

**Background:**

Protein kinase inhibitors are central drugs in precision oncology, but cell-line response to these agents is affected by lineage and molecular context rather than target identity alone. Many drug-response models report optimistic internal performance; stricter evaluation requires held-out drugs, independent screens and cross-platform validation. This study evaluates a focused, auditable pharmacogenomic modelling framework for kinase-inhibitor response prediction.

**Methods:**

Protein kinase inhibitors were curated from PKIDB and matched to GDSC release 8.5 response data and DepMap 26Q1 molecular profiles. The full matched screens contained GDSC2 (40,403 drug-cell line pairs; 42 drugs; 945 models), GDSC1 (40,900 pairs; 48 drugs; 945 models) and PRISM secondary screen data (7,090 pairs; 23 drugs; 336 models). GDSC2-to-GDSC1 external testing was restricted to compounds shared by both GDSC releases (23,340 GDSC2 training pairs and 22,570 GDSC1 test pairs; 25 drugs). Models were evaluated using cell-line cross-validation, drug cross-validation, independent-screen validation, continuous-response prediction and pathway/drug subgroup analysis.

**Results:**

In the GDSC1 shared-drug external set, molecular-summary models achieved ROC AUC 0.706 for GDSC AUC-defined sensitivity and 0.692 for LN_IC50-defined sensitivity. Continuous-response validation in the same GDSC1 test set produced Spearman correlations of 0.453 for AUC and 0.457 for LN_IC50, with continuous-derived sensitivity ROC AUCs of 0.703 and 0.710. Molecular summaries improved external ROC AUC over drug-lineage context by 0.052 for AUC sensitivity and 0.046 for LN_IC50 sensitivity. ERK/MAPK signalling showed the strongest pathway-level signal, with ROC AUC 0.825 for AUC sensitivity and 0.780 for LN_IC50 sensitivity. PRISM cross-platform validation was more modest (best ROC AUC 0.597), consistent with assay and drug-overlap differences.

**Conclusion:**

PKIDB-informed molecular profiling improves externally reproducible kinase-inhibitor response modelling across public cancer cell-line screens, especially for ERK/MAPK inhibitors. These findings support a pharmacogenomic modelling and validation study rather than direct patient-level or experimental validation.

## 1 Introduction

Protein kinase inhibitors occupy a central position in precision oncology because they target actionable signalling dependencies across multiple tumour lineages. However, response to a kinase inhibitor is rarely explained by nominal target annotation alone. Cancer lineage, transcriptional state, copy-number change, mutation status and genetic dependency can all modify the relationship between compound exposure and observed viability. For this reason, public pharmacogenomic resources provide a useful test bed for evaluating whether molecular profiles add reproducible predictive information beyond drug descriptors and lineage labels (Yang et al., 2013; Iorio et al., 2016; Garnett et al., 2012). Related cell-line screening resources, including CCLE and CTRP, established the wider basis for comparing lineage, genetic context and small-molecule response (Barretina et al., 2012; Basu et al., 2013). Integrative resources such as PharmacoDB and PharmacoGx further support cross-study pharmacogenomic analysis (Smirnov et al., 2018; Smirnov et al., 2016).

A major problem in computational drug-response studies is the tendency to report performance from random or row-wise splits within a single screen. Such evaluations are useful during model development, but they do not fully address generalisation across drugs, cell lines or independent assays, and previous work has shown that pharmacogenomic screens can differ materially across platforms (Haibe-Kains et al., 2013; Xia et al., 2022). Early machine-learning drug-response studies helped define the basic feature-integration problem, but they were usually less explicit about cross-screen validation (Menden et al., 2013). Recent reviews have also stressed dataset inconsistency and generalisation limits in drug-response prediction (Zhang X. et al., 2026; Tran and Klau, 2026). Newer models increasingly use pathway-guided graphs and heterogeneous networks (Yoon and Lee, 2026; Zhang J. et al., 2026). Network-based and gene-regulatory representations have extended this idea to subclonal or regulatory structure (Kim et al., 2026; Xie et al., 2024). Transfer-learning and single-cell extensions have broadened the field, but they still depend on transparent dataset definition and validation design (Meng et al., 2025; Zhou et al., 2025). For journal-level evidence, the validation chain should therefore separate cell-line cross-validation, drug cross-validation, independent GDSC validation and more distant cross-platform validation.

This study focuses on a defensible public-data question: whether PKIDB-informed kinase-inhibitor curation and DepMap molecular context improve reproducible response prediction in GDSC and PRISM pharmacogenomic screens (Carles et al., 2018; Corsello et al., 2020). The kinase-specific framing is also supported by recent work on kinase-inhibitor profiling, kinase-activity inference and kinome-wide affinity prediction (Wu et al., 2024; Zhang et al., 2024; Schifferstein et al., 2024). This framing gives the article a clear evidence route: public database construction, model validation, molecular-feature gain, pathway-level interpretation and transparent limitations.

This study therefore does not attempt to claim a universal pan- drug predictor. Instead, it asks a narrower and more testable question. If kinase-inhibitor response is partly governed by pathway state and dependency context, then molecular summaries should improve prediction when the same drug class is evaluated in an independent screen. A positive result under this design is more informative than a high score from a single random split, because the model must carry signal across release-specific response measurements and cell-line matching. Conversely, weaker PRISM performance would not invalidate the GDSC result; it would define the boundary between same-resource external validation and a more distant assay platform.

## 2 Methods

### 2.1 Data sources and harmonisation

Protein kinase inhibitors were defined using the PKIDB download dated 15 April 2025 (Carles et al., 2018). Drug names and compound annotations were harmonised against GDSC release 8.5 (Yang et al., 2013; Iorio et al., 2016; Garnett et al., 2012). GDSC2 was used as the primary development screen, and GDSC1 was retained as an independent external validation screen. DepMap 26Q1 molecular profiles were matched at the cell-line model level, including expression, copy-number and CRISPR dependency features derived from the Cancer Dependency Map resource (Tsherniak et al., 2017; Behan et al., 2019; Dempster et al., 2021). The analysed kinase-inhibitor set was restricted to PKIDB-matched response records rather than to the broader GDSC screened-compound catalogue. GDSC2-to-GDSC1 external validation used only drugs present in both releases, giving 23,340 GDSC2 training pairs and 22,570 GDSC1 test pairs for 25 shared drugs. PRISM secondary screen data were used after shared-drug and shared-model matching and were interpreted as cross-platform supporting evidence rather than as equivalent to GDSC validation (Corsello et al., 2020). The datasets and their respective roles in model development and validation are summarised in Table 1.

**TABLE 1:** Public datasets used in the analysis.

Dataset	Role	Full pairs	Full drugs	Models	Modelled validation subset
GDSC2 + DepMap	Training/internal validation	40,403	42	945	CV plus shared-drug external training subsets
GDSC1 + DepMap	External validation	40,900	48	945	22,570 test pairs for 25 shared drugs
PRISM + DepMap	Cross-platform validation	7,090	23	336	7,090 cross-platform test pairs

The harmonisation step was treated as part of the scientific design rather than as a file-processing detail. Kinase inhibitors can appear under alternative spellings, salt forms or abbreviated names across resources, and public screens do not always preserve a one-to-one compound mapping. The working dataset was therefore built only after drug names, cell-line identifiers and DepMap model identifiers were made consistent. Records without a usable response endpoint or without matched molecular context were not carried into modelling. This conservative restriction reduces the apparent sample space, but it prevents release-level catalogue counts from being confused with the number of compounds that actually support a reproducible modelling experiment.

### 2.2 Response endpoints

Two endpoint families were analysed. First, binary sensitivity labels were defined within each drug by comparing GDSC AUC or LN_IC50 values with the drug-specific median. This endpoint asks whether a model can rank cell lines as relatively sensitive or resistant for a given drug. Second, continuous drug-level z-score responses were modelled for AUC and LN_IC50. Continuous models were evaluated by Spearman correlation, Pearson correlation, RMSE, MAE and the ROC AUC obtained after converting continuous predictions back into drug-median sensitivity labels, recognising that alternative dose-response metrics can change biological interpretation (Fallahi-Sichani et al., 2013).

Using both binary and continuous endpoints reduces dependence on a single response definition. The median-based binary endpoint is useful for classification because it balances sensitivity labels within each drug and avoids comparing raw response thresholds across compounds with different potency ranges. The continuous endpoint retains more response variation and is better suited for asking whether predicted rankings track measured dose-response values within the external screen. Agreement between these two endpoint families was interpreted as stronger evidence than either endpoint alone, while disagreement would indicate that a model may rank broad sensitive/resistant groups without accurately preserving continuous response gradients.

### 2.3 Feature sets and models

Three feature sets were compared. Drug-lineage context included compound descriptors and cancer-lineage annotation. Molecular-summary features added target and pathway expression, copy-number, CRISPR dependency and mutation summaries from DepMap. A broader gene-panel representation retained additional kinase-relevant molecular variables. Random forest, ExtraTrees, LightGBM, XGBoost and regularised linear models were evaluated according to endpoint type. Tree-ensemble methods were used as robust tabular baselines (Breiman, 2001; Geurts et al., 2006; Ke et al., 2017), and implementation followed standard machine-learning tooling (Chen and Guestrin, 2016; Pedregosa et al., 2011). Model evaluation prioritised performance in the independent GDSC1 external validation set, while internal cross-validation was used primarily for model development and comparison.

The feature sets were structured to evaluate the incremental contribution of molecular information beyond drug and lineage context. Drug-lineage features assessed the predictive information provided by compound descriptors and cancer-lineage annotation alone, whereas molecular-summary features incorporated shared DepMap-derived biological information while maintaining a relatively compact feature representation. The broader gene-panel representation served as a higher-dimensional comparison to assess whether additional kinase-relevant molecular variables further improved predictive performance. This framework enabled evaluation of whether predictive gains were attributable to biologically informed target, pathway, and dependency features rather than solely to increased feature dimensionality.

### 2.4 Validation and subgroup analysis

Validation was performed at four levels: cell-line cross-validation within GDSC2, drug cross-validation within GDSC2, GDSC2-to-GDSC1 external validation and PRISM cross-platform validation. Bootstrap confidence intervals were calculated for the external performance difference between molecular-summary features and drug-lineage context. Pathway, drug and lineage subgroup analyses were performed to evaluate performance variation across biological and pharmacological strata.

In cell-line cross-validation, training and testing folds contained different cell lines while retaining the same drug set and response resource. In drug cross-validation, drugs were split across folds to evaluate generalisation to held-out compounds within GDSC2. In GDSC2-to-GDSC1 external validation, models trained in GDSC2 were evaluated on shared drugs and cell lines in GDSC1. In PRISM cross-platform validation, trained models were evaluated on the subset of PRISM drug-cell line pairs that could be matched to PKIDB drug annotations and DepMap model identifiers.

### 2.5 Sensitivity and *post hoc* error analyses

Sensitivity and *post hoc* error analyses were performed to accompany the external and cross-platform validation experiments. For the PRISM analysis, cross-platform validation performance was first compared with GDSC1 external validation. Exact shared GDSC1-PRISM pairs were defined as observations with matched PKIDB drug annotations and matched DepMap model identifiers in both datasets. Within these exact shared pairs, we calculated median-based sensitivity-label agreement, discordant label directions, the distributional difference between PRISM AUC and GDSC1 AUC, drug-level discordance fractions, log10 dose-range width and pathway-level GDSC1-PRISM Spearman concordance.

For endpoint sensitivity analysis, alternative response labels were generated in the GDSC1 external validation set. Fixed-threshold labels included IC50 < 1 μM, corresponding to LN_IC50 < 0, and AUC cutoffs of 0.80, 0.85 and 0.90. Drug-specific waterfall-inflection thresholds were also derived separately for AUC and LN_IC50 using each drug’s response distribution. For each alternative endpoint, we calculated agreement with the original drug-median labels, positive-class fraction, ROC AUC and average precision. Positive-class fraction was reported together with average precision because precision-recall metrics are affected by class prevalence. The distributions of drug-specific waterfall thresholds were summarized and compared with the fixed AUC and IC50 reference thresholds. Median-based and waterfall-derived labels were further cross-tabulated to evaluate how the stricter waterfall sensitivity definitions related to the original median-based labels.

For pathway-focused *post hoc* analysis, ERK/MAPK and RTK signalling were compared using feature-set ablation, ROC AUC gain from molecular-summary features relative to drug-lineage features, putative target-count distributions, Spearman associations between pathway- or target-level expression and CRISPR dependency summaries and drug response, grouped feature-importance values and drug-level ROC AUC distributions.

## 3 Results

### 3.1 Study design and dataset landscape

The study workflow is shown in Figure 1. The pipeline begins with public response and molecular-profile data, proceeds through drug-name harmonisation and feature construction, and then separates internal validation from independent-screen testing.

**FIGURE 1:**
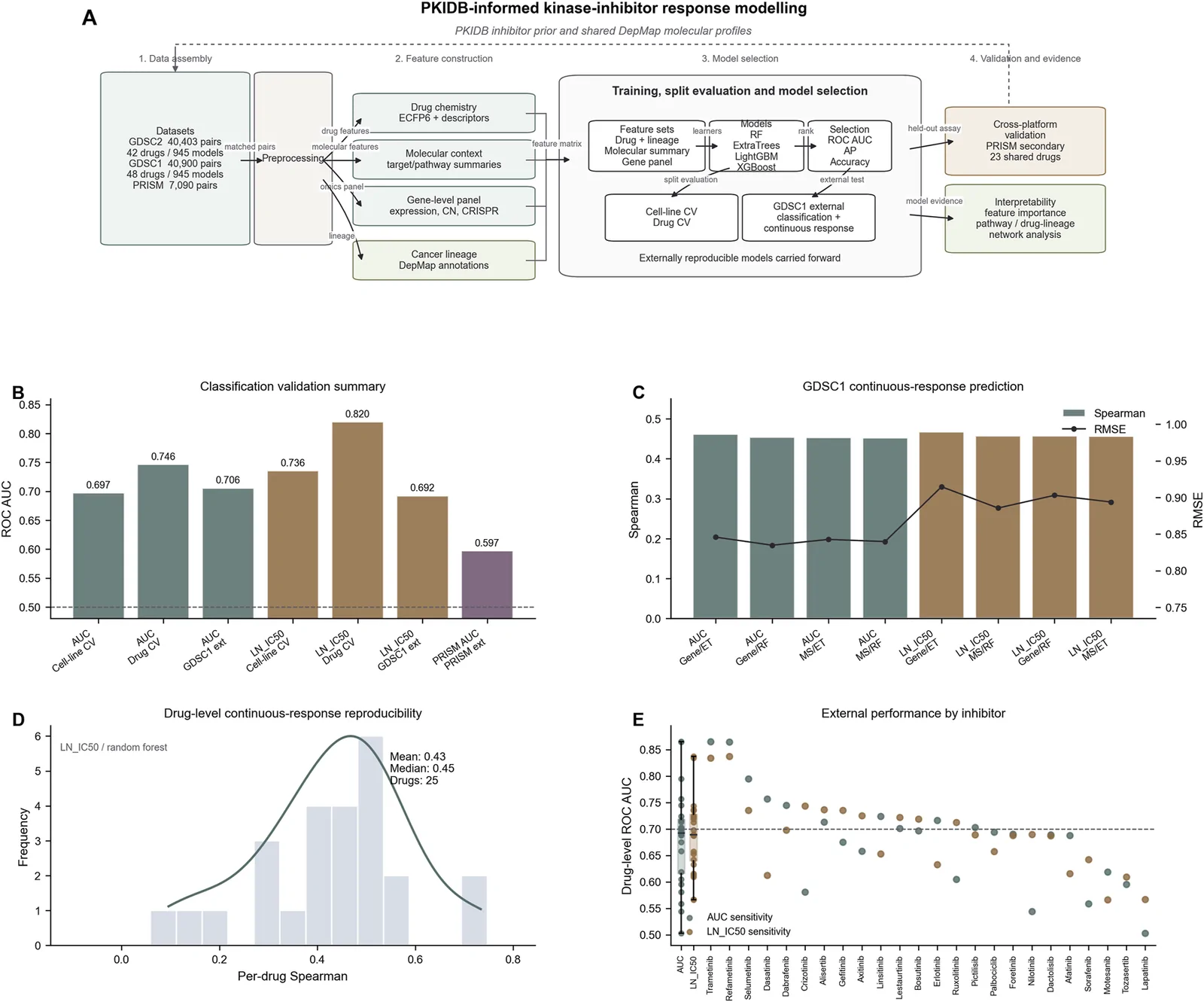
Study design and modelling framework. **(A)** Overall workflow for PKIDB-informed kinase-inhibitor response modelling, including data assembly, preprocessing, feature construction, model selection and validation. **(B)** Summary of binary sensitivity classification performance across internal cell-line cross-validation, drug cross-validation, GDSC1 external validation and PRISM cross-platform validation. **(C)** GDSC1 continuous-response prediction performance for AUC and LN_IC50 models, showing Spearman correlation and RMSE across model classes. **(D)** Drug-level continuous-response reproducibility, summarised as the distribution of per-drug Spearman correlations. **(E)** Drug-level external ROC AUC values for representative inhibitors under AUC- and LN_IC50-defined sensitivity endpoints; the left-side boxplots summarise the endpoint-specific drug-level ROC AUC distributions.

At the data-construction stage, the effective analysis unit was a drug-cell-line pair with three linked components: a curated kinase-inhibitor identity, a measured response value and a matched DepMap molecular profile. The model was not trained on all screened compounds in a release, and it was not evaluated on records lacking molecular context. Instead, every reported validation result was tied to the matched subset that could support the relevant comparison. Figure 1 summarises the flow from public catalogues to model-ready datasets, rather than a simple count of all compounds ever screened by GDSC or PRISM.

The dataset landscape in Figure 2 shows that GDSC2 and GDSC1 provide closely related but non-identical kinase-inhibitor response spaces, whereas PRISM provides a smaller and more distant assay platform. This structure supports a tiered evidence interpretation: GDSC1 is the main external validation screen, and PRISM is a stricter cross-platform stress test.

**FIGURE 2:**
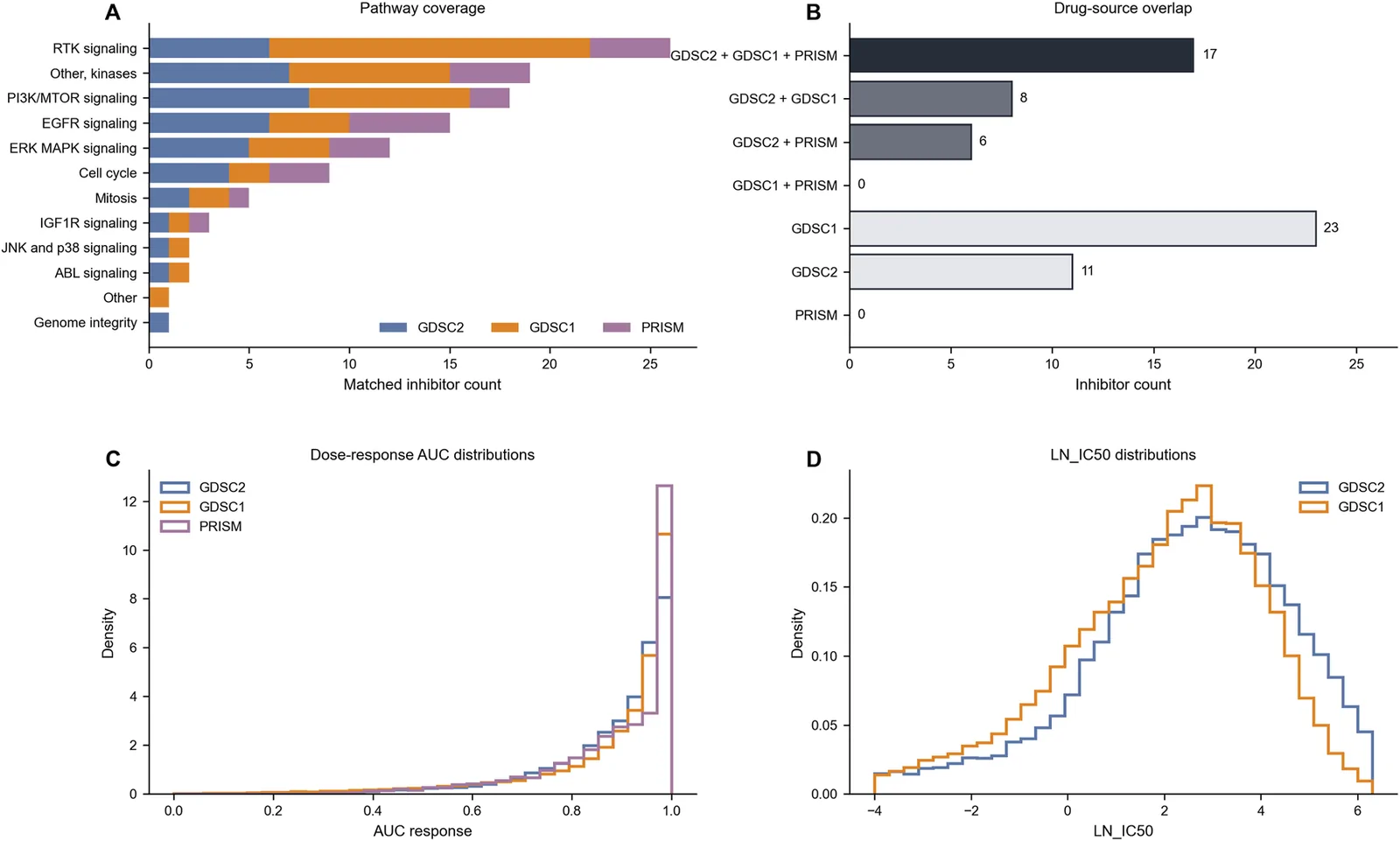
Dataset landscape and drug overlap. **(A)** Matched inhibitor coverage across kinase pathway groups in GDSC2, GDSC1 and PRISM. **(B)** Exclusive drug-source overlap combinations among GDSC2, GDSC1 and PRISM after PKIDB and DepMap matching, including the GDSC1 + PRISM combination. **(C)** Distribution of dose-response AUC values across the matched GDSC2, GDSC1 and PRISM datasets. **(D)** Distribution of LN_IC50 values in GDSC2 and GDSC1.

This structure also explains why the paper reports full matched-screen size separately from the external validation subset. The full GDSC2 and GDSC1 tables describe the available kinase-inhibitor response landscape after PKIDB and DepMap matching. The shared-drug GDSC2-to-GDSC1 subset describes the part of that landscape that can support direct external validation. PRISM is smaller because the secondary screen shares fewer kinase inhibitors and fewer matched models under the same inclusion rules. These differences are important for interpretation: a model can be technically trained on a larger resource while only a smaller, clearly defined fraction is appropriate for external testing.

Figures 1, 2 are presented sequentially to delineate this distinction before model performance is evaluated. Figure 1 outlines the experimental framework, including the curated kinase-inhibitor prior, matched molecular profiles, model selection, and external validation. Figure 2 shows the corresponding dataset structure, demonstrating that the three public screens contribute different but complementary evidence. Together, these figures provide the data context for the subsequent ROC AUC analyses and clarify the datasets used for model development, external testing, and cross-platform evaluation.

### 3.2 Classification validation

Binary sensitivity models showed moderate but reproducible external performance. In the GDSC1 shared-drug external test set (22,570 pairs; 25 drugs; 945 models), the best molecular-summary model reached ROC AUC 0.706 for AUC-defined sensitivity and 0.692 for LN_IC50-defined sensitivity. Drug cross-validation performance was higher, particularly for LN_IC50 sensitivity, but it should be interpreted as within-GDSC2 drug generalisation rather than as independent-screen evidence (Figure 3).

**FIGURE 3:**
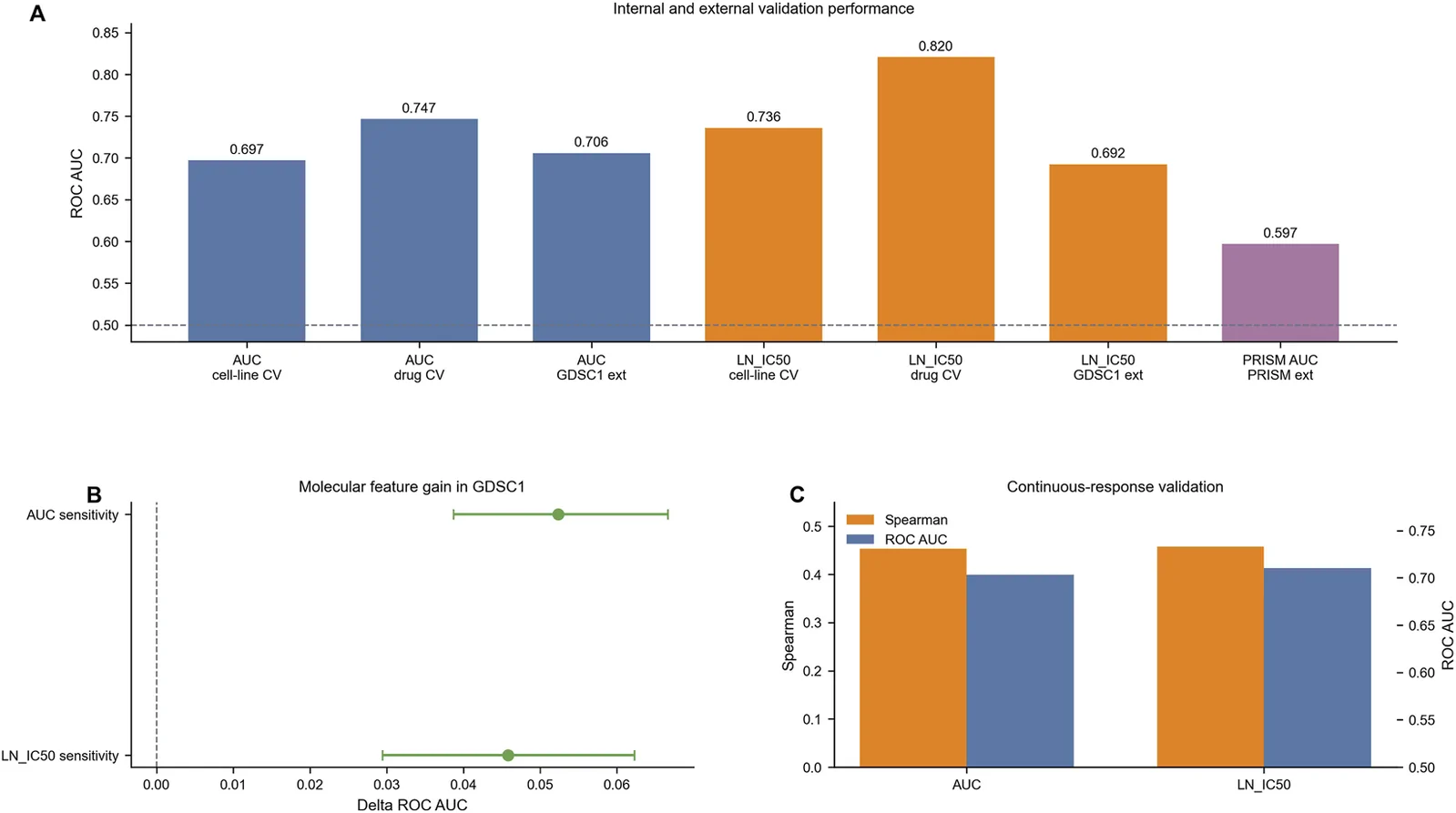
Model validation summary. **(A)** Internal and external binary-classification performance across cell-line cross-validation, drug cross-validation, GDSC1 external validation and PRISM cross-platform validation. **(B)** Bootstrap-estimated ROC AUC gain of molecular-summary features over drug-lineage context in the GDSC1 external validation set. **(C)** Continuous-response validation in GDSC1, showing Spearman correlation and ROC AUC after converting continuous predictions back into drug-median sensitivity labels.

The external AUC values are not presented as near-clinical diagnostic accuracy. Their value is that they remain above the drug-lineage baselines after the model is transferred from GDSC2 to GDSC1. In a noisy pharmacogenomic setting, this is a more relevant signal than a high internal score, because response measurements are affected by screening protocol, curve fitting, drug concentration range and cell-line annotation. The same pattern across both AUC-defined and LN_IC50-defined sensitivity indicates that the external signal is not restricted to one response metric.

Table 2 clarifies the role of each validation setting. Cell-line cross-validation is a development check, drug cross-validation tests held-out compounds within GDSC2, and the GDSC1 rows provide the main evidence for external reproducibility. The paired AUC and LN_IC50 rows show that the same molecular-summary feature block remains useful under two response definitions.

**TABLE 2:** Best binary sensitivity models by endpoint and validation setting.

Endpoint	Validation	Feature set	Model	AUC	AP
AUC sensitivity	Cell-line CV	Molecular summary	extra_trees	0.697	0.697
AUC sensitivity	Drug CV	Gene panel	extra_trees	0.747	0.738
AUC sensitivity	GDSC1 external	Molecular summary	random_forest	0.706	0.696
LN_IC50 sensitivity	Cell-line CV	Molecular summary	extra_trees	0.736	0.745
LN_IC50 sensitivity	Drug CV	Gene panel	extra_trees	0.820	0.821
LN_IC50 sensitivity	GDSC1 external	Molecular summary	random_forest	0.692	0.675

### 3.3 External continuous-response prediction

Continuous-response validation supported the classification results. In the same GDSC1 shared-drug test set, molecular-summary models achieved Spearman correlations of 0.453 for AUC and 0.457 for LN_IC50. When continuous predictions were converted back into drug-median sensitivity labels, ROC AUCs were 0.703 and 0.710, respectively.

Molecular-summary features also improved external classification performance over drug-lineage context. The bootstrap mean improvement in ROC AUC was 0.052 for AUC sensitivity (95% CI 0.039–0.067) and 0.046 for LN_IC50 sensitivity (95% CI 0.029–0.062). These intervals excluded zero, supporting the incremental contribution of shared molecular profiles to external predictive performance.

The bootstrap improvement should be interpreted in the context of the prediction task. The models ranked cell-line sensitivity across compounds and tumour lineages in an independent pharmacogenomic screen rather than predicting patient-level treatment response. Under this design, the observed ROC AUC gains support the added predictive value of the shared molecular summaries, which were derived independently of the target screen. The continuous-derived ROC AUCs of 0.703 and 0.710, together with the fixed-threshold sensitivity results, provide additional evidence that the external classification results were not solely driven by median-thresholding.

Figure 4 Combines several views of the same external validation problem. The ROC and precision-recall panels show discrimination under drug-median sensitivity labels. The calibration panel evaluates the agreement between predicted probabilities and observed sensitive fractions beyond discrimination alone. The continuous-response panel retains the underlying response scale and provides a complementary assessment beyond binary classification.

**FIGURE 4:**
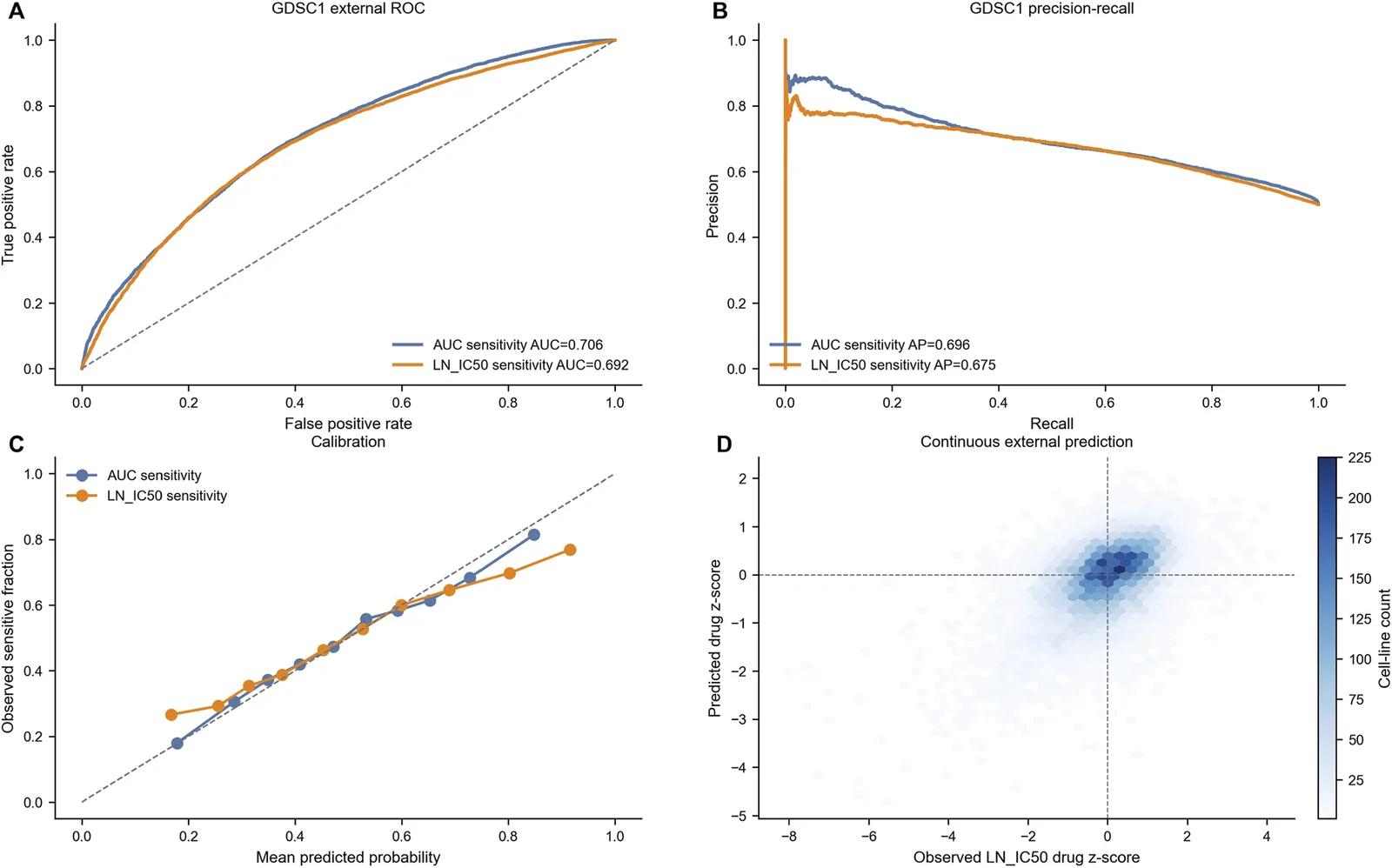
GDSC1 external validation. **(A)** ROC curves for AUC- and LN_IC50-defined sensitivity labels in the GDSC1 external validation set. **(B)** Precision-recall curves for the same external validation tasks. **(C)** Calibration curves comparing predicted sensitivity probabilities with observed sensitive fractions. **(D)** Continuous external prediction for LN_IC50 drug z-scores, shown as observed versus predicted response density.

### 3.4 Pathway and drug-level reproducibility

Pathway analysis identified ERK/MAPK signalling as the clearest reproducible subgroup (Table 3; Figure 5). For this pathway, GDSC1 external ROC AUC was 0.825 for AUC sensitivity and 0.780 for LN_IC50 sensitivity. Drug-level analysis was concordant: Trametinib and Refametinib were among the strongest external examples, with ROC AUC 0.865 and 0.865, respectively (Table 4; Figure 5).

**TABLE 3:** Leading pathway-level external validation results.

Endpoint	Pathway	Rows	Drugs	ROC AUC	AP
AUC sensitivity	ERK MAPK signaling	5,171	4	0.825	0.794
AUC sensitivity	IGF1R signaling	843	1	0.724	0.701
AUC sensitivity	PI3K/MTOR signaling	2,641	2	0.698	0.675
AUC sensitivity	Cell cycle	867	1	0.694	0.631
AUC sensitivity	EGFR signaling	3,070	3	0.682	0.708
AUC sensitivity	Mitosis	1,247	2	0.672	0.673
AUC sensitivity	Other, kinases	2,708	3	0.671	0.664
AUC sensitivity	RTK signaling	4,223	7	0.634	0.624

**FIGURE 5:**
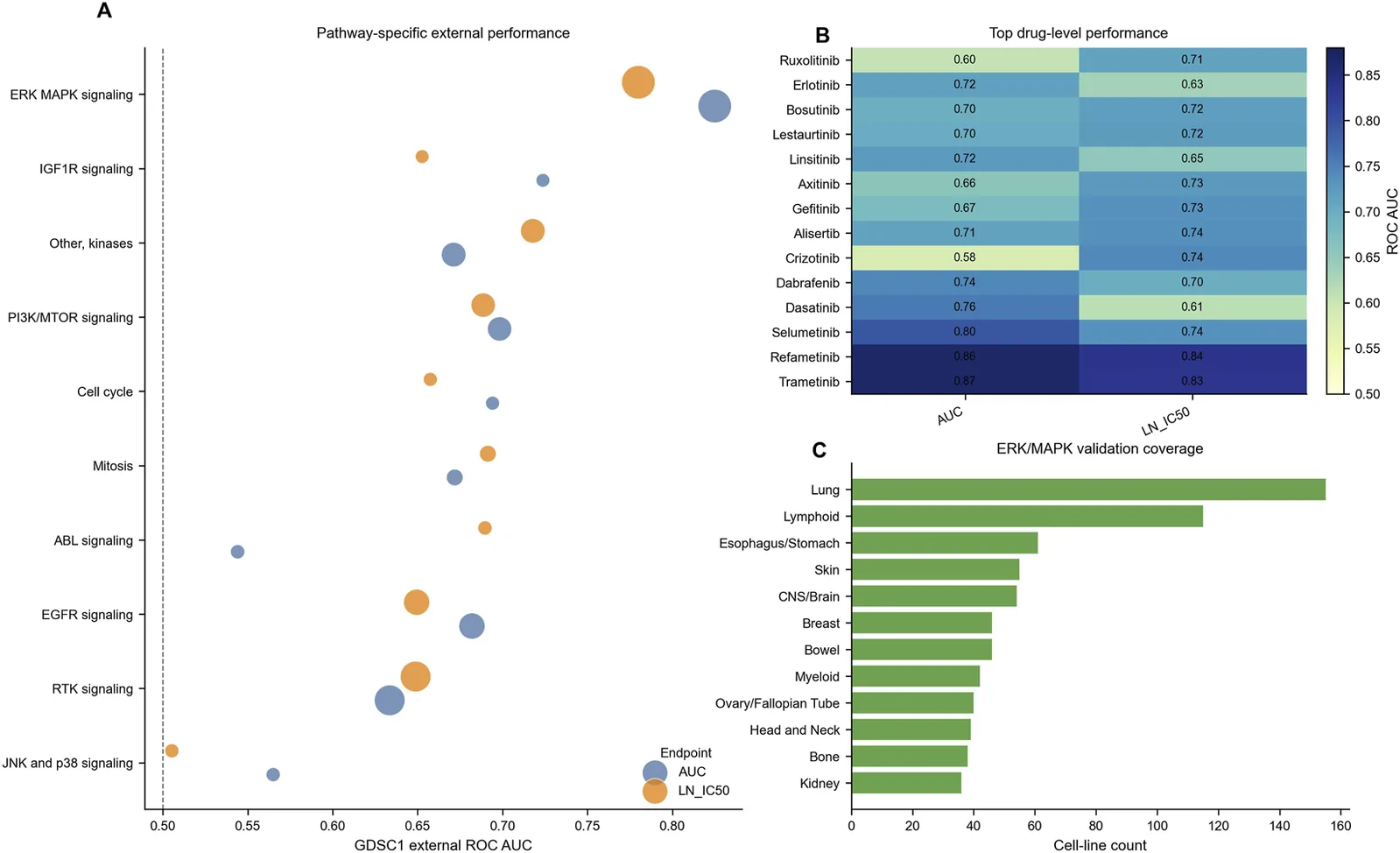
Pathway and drug subgroup performance. **(A)** Pathway-specific GDSC1 external ROC AUC values for AUC- and LN_IC50-defined sensitivity; point size reflects the number of evaluable observations. **(B)** Top drug-level external ROC AUC values for representative inhibitors under AUC and LN_IC50 sensitivity endpoints. **(C)** Cell-line coverage of the ERK/MAPK validation subset across tumour lineages.

**TABLE 4:** Top drug-level GDSC1 external validation examples for AUC-defined sensitivity.

Drug	Endpoint	Rows	ROC AUC	AP
Trametinib	AUC sensitivity	860	0.865	0.836
Refametinib	AUC sensitivity	1730	0.865	0.829
Selumetinib	AUC sensitivity	1742	0.795	0.758
Dasatinib	AUC sensitivity	378	0.757	0.790
Dabrafenib	AUC sensitivity	839	0.745	0.768

These findings indicate that pathway-level reproducibility was not uniform across kinase groups. The ERK/MAPK subgroup showed the most consistent external signal, whereas RTK signalling was less completely captured by compact target- and pathway-level summaries. This pathway-level disparity supports a kinase-focused evaluation rather than relying only on a broad all-compound benchmark. Feature-importance and pathway-focused analyses further connected the ERK/MAPK performance signal to target and pathway molecular profiles (Figure 6).

**FIGURE 6:**
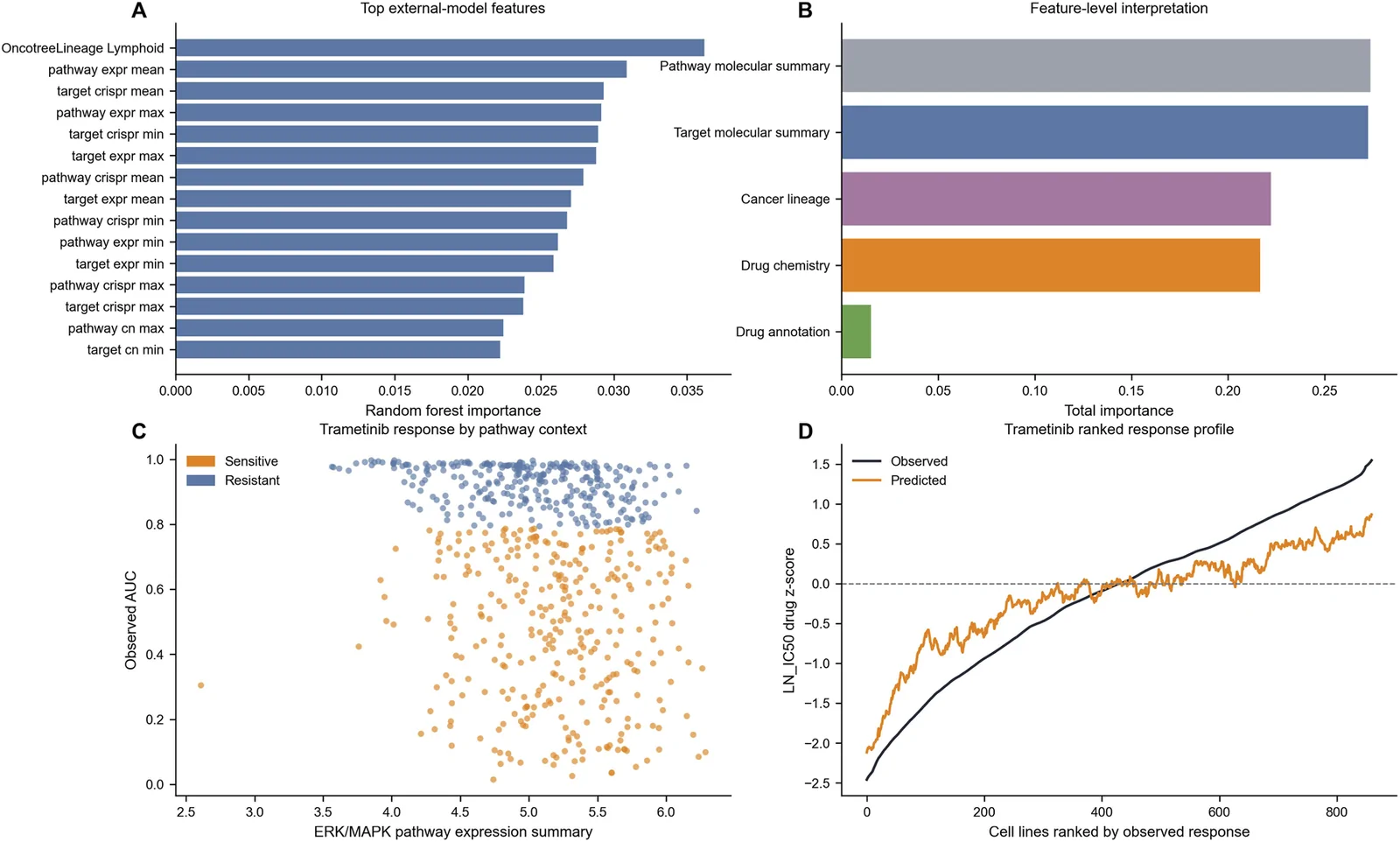
Interpretability and ERK/MAPK case study. **(A)** Top random-forest feature importances in the GDSC1 external model. **(B)** Aggregated feature importance by category, comparing pathway molecular summaries, target molecular summaries, cancer lineage, drug chemistry and drug annotation. **(C)** Trametinib response pattern in relation to ERK/MAPK pathway expression summary, with points coloured by sensitive or resistant status. **(D)** Ranked Trametinib response profile comparing observed and predicted LN_IC50 drug z-scores across cell lines.

### 3.5 PRISM cross-platform validation and network view

PRISM validation was treated as supplementary cross-platform evidence because the assay platform and shared-drug set differ from GDSC. The best PRISM model reached ROC AUC 0.597, average precision 0.608 and accuracy 0.567. This lower performance is consistent with the greater platform differences and indicates more limited cross-platform transferability than observed in the GDSC validation setting.

The PRISM analysis further assessed model transfer across fewer shared drugs and a different screening technology. Its lower performance indicates that, although molecular profiling showed reproducible predictive value within the GDSC validation chain, cross-platform transfer remained limited. Separately, the drug-lineage response network summarises externally supported GDSC1 drug-lineage associations and highlights where robust kinase-inhibitor response patterns were concentrated across drugs, pathways and tumour lineages (Figure 7).

**FIGURE 7:**
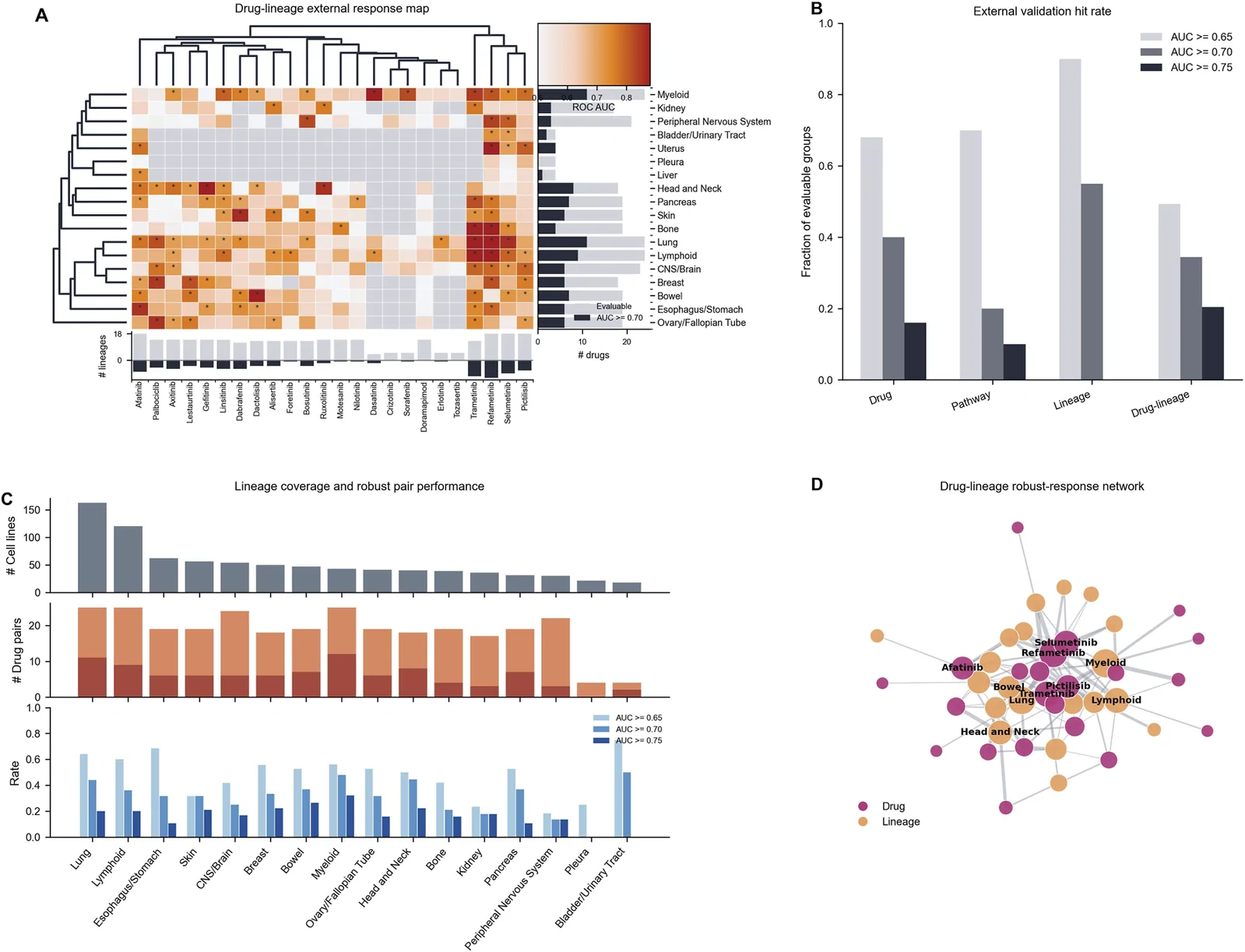
Drug-lineage response network. **(A)** Heatmap of externally supported drug-lineage response associations, with clustering of drugs and tumour lineages. **(B)** Fraction of evaluable drug, pathway, lineage and drug-lineage groups meeting external ROC AUC thresholds of 0.65, 0.70 and 0.75. **(C)** Lineage-level coverage and robust drug-lineage pair performance, including cell-line counts, evaluable drug pairs and threshold-specific hit rates. **(D)** Drug-lineage robust-response network linking inhibitors and tumour lineages with externally supported response patterns.

### 3.6 Sensitivity and *post hoc* error analyses

Sensitivity and *post hoc* analyses were performed to evaluate the robustness of the external validation results and to further examine the main sources of performance variation.

First, endpoint sensitivity analysis showed that the external predictive signal was not dependent on drug-median binarization alone. Alternative endpoint definitions, including IC50 < 1 μM, fixed AUC thresholds and drug-specific waterfall-inflection thresholds, showed only moderate agreement with drug-median labels, with agreement values ranging from approximately 0.63–0.74. This confirmed that endpoint choice affected sensitivity/resistance assignment. However, the model retained above-random discrimination under all alternative endpoint definitions, with all ROC AUC values remaining above 0.6. Fixed-threshold and waterfall-derived labels introduced substantial class-balance shifts, with positive-class fractions varying across endpoint definitions; therefore, average precision was interpreted together with class prevalence. Drug-specific waterfall thresholds varied widely for both AUC and LN_IC50, indicating that a single fixed threshold could not fully represent all kinase inhibitors. The median-versus-waterfall label matrix further showed that AUC waterfall-sensitive observations formed a stricter subset of median-sensitive observations in this analysis. These results indicate that the median endpoint is suitable for balanced pharmacogenomic benchmarking, whereas waterfall-derived endpoints provide stricter sensitivity checks rather than direct clinical response criteria (Supplementary Figure S1).

Second, pathway-focused *post hoc* analysis showed that the lower RTK performance reflected more than a numerical subgroup difference. Compared with ERK/MAPK signalling, RTK signalling showed a smaller ROC AUC gain from molecular-summary features over drug-lineage features, and RTK-associated inhibitors had broader putative target spectra. Molecular-summary response associations also differed between pathways: ERK/MAPK showed a stronger pathway-level CRISPR dependency signal, whereas RTK showed weaker pathway-level dependency signal and a less coherent molecular-summary pattern. At the drug level, RTK-associated inhibitors showed greater performance heterogeneity than ERK/MAPK inhibitors. These findings suggest that compact target- and pathway-level summaries captured ERK/MAPK inhibitor response more effectively than RTK inhibitor response, while the weaker RTK results were consistent with target redundancy, polypharmacology and drug-specific variability (Supplementary Figure S2).

Third, PRISM error analysis showed that the cross-platform performance decrease was accompanied by response discordance between GDSC1 and PRISM. Median-based sensitivity labels agreed in only 59.6% of exact shared GDSC1-PRISM drug-model pairs, with discordance occurring in both directions. PRISM AUC values showed a modest shift relative to GDSC1 AUC values, and PRISM used a wider dose-range context. Drug-level discordance was concentrated in several kinase inhibitors, including RTK-associated drugs such as Sorafenib, Foretinib, Motesanib and Dasatinib. Pathway-level concordance was also stronger for ERK/MAPK than for RTK signalling. These results support interpreting PRISM as a cross-platform portability stress test rather than a direct replication of the GDSC1 validation tier (Supplementary Figure S3).

## 4 Discussion

The study provides a focused evaluation of kinase-inhibitor response modelling. Its main result is the observation that DepMap molecular context improves response prediction under independent GDSC validation, rather than the achievement of high internal AUC values. This point is important because recent methodological studies have substantially expanded the range of modelling approaches. Pathway- and graph-based models now provide more interpretable prediction frameworks (Yoon and Lee, 2026; Zhang J. et al., 2026; Xiang et al., 2025). Drug-specific network feature selection and web-based interpretable predictors similarly emphasise biologically interpretable modelling (Pak et al., 2024; Cadow et al., 2020). Transfer-learning and single-cell models have been developed to bridge cell-line screens with patient or single-cell settings (Meng et al., 2025; Zhou et al., 2025; Sinha et al., 2024). Other recent work has explored contrastive representations, multi-omics integration and clinically optimised pharmacogenomic discovery (Lawrence et al., 2024; Saranya and Vimina, 2024; Truesdell et al., 2024). SMILES-based and general deep-learning response predictors further expand the methodological landscape (Abbasi et al., 2024; Shahzad et al., 2024). Recent studies of general drug-sensitivity principles, drug-combination transfer and tumour-type-focused prediction reinforce the need to define the validation target clearly (Carli et al., 2025; Guo et al., 2026; Xerxa et al., 2026). Robust dataset splitting and external validation therefore remain central to establishing the biological credibility and generalisability of drug-response models.

The ERK/MAPK subgroup gives the strongest biological support. Trametinib, Refametinib and related inhibitors target MAPK-pathway signalling, and their high external performance suggests that target and pathway molecular summaries capture information beyond drug identity and lineage. This pathway-level interpretation gives the model a clearer mechanistic basis than an undifferentiated pan-cancer benchmark. The same logic is consistent with targeted-class studies in which cellular architecture or pathway state shapes response and resistance to a specific inhibitor class (Park et al., 2024).

The RTK subgroup defines an important boundary of the current feature-engineering strategy. In the pathway-focused *post hoc* analysis, RTK signalling showed a smaller gain from molecular-summary features than ERK/MAPK signalling, and RTK-associated inhibitors had broader putative target spectra, weaker pathway-level CRISPR dependency signal and greater drug-level heterogeneity. Unlike ERK/MAPK inhibitors, which often target a more concentrated downstream signalling axis, RTK inhibitors act across a diverse receptor-family network, including EGFR, FGFR, VEGFR, MET, AXL, PDGFR and related kinases. Dependence on these receptors can vary substantially across tumour lineages and cell-line contexts. This interpretation is consistent with previous studies showing that resistance to RTK inhibition can arise through activation of alternative RTKs, bypass signalling and downstream pathway re-engagement (Niederst and Engelman, 2013; Ware et al., 2013). In addition, inhibition of one receptor can be compensated by other RTKs or by downstream bypass signalling through PI3K/AKT or RAS/MAPK branches. Many RTK inhibitors are also polypharmacologic, so response may be driven by secondary or off-target kinase effects that are not fully represented by compact PKIDB-derived target summaries (Knight et al., 2010; Ma et al., 2018). These mechanisms can weaken the relationship between aggregate target/pathway features and observed drug response. Mean, maximum and minimum summaries of expression, copy number and CRISPR dependency are interpretable and low-dimensional, but they may dilute the specific receptor dependency, mutation state, ligand context, phosphorylation activity or feedback regulation that determines RTK inhibitor sensitivity. This limitation is consistent with the broader kinase-drug literature, where inhibitor selectivity, target redundancy and context-dependent signalling remain central challenges for predicting response (Attwood et al., 2021). RTK signalling therefore represents a biologically plausible limitation of the current interpretable summary-feature approach and a priority area for richer mechanistic modelling.

The PRISM analysis further defines the boundary of the current evidence. Cross-platform performance was lower than GDSC1 external performance, and the expanded exact-pair error analysis showed that this decrease was accompanied by median-label discordance, modest response-distribution shift and different dose-range context between GDSC1 and PRISM. The discordance was also drug-dependent, with several multi-target or RTK-associated inhibitors showing higher cross-platform label instability. This lower portability is consistent with differences in assay technology, dose-response processing, barcode-based viability readout, dose-range design and pathway-dependent response heterogeneity. PRISM should therefore be interpreted as a cross-platform portability stress test rather than as a direct replication of the GDSC1 validation tier.

The endpoint definition provides another boundary for interpretation. Intra-drug median binarization is useful for pharmacogenomic benchmarking because it balances sensitivity labels within each drug, but it does not define a clinical response threshold. The expanded endpoint sensitivity analysis showed that the external signal was retained under IC50 < 1 μM, fixed AUC cutoffs and drug-specific waterfall-inflection thresholds, supporting the view that the result was not driven only by median-thresholding. However, these alternative endpoints introduced class-balance shifts, and the waterfall analyses showed that drug-specific sensitivity cutoffs varied across inhibitors. Therefore, the median endpoint is suitable for balanced benchmarking, whereas waterfall-derived endpoints provide stricter sensitivity checks. Because all endpoint definitions were derived from public 2D cell-line screens, they should be interpreted as biological benchmarking checks rather than patient-response criteria.

Taken together, these results define the appropriate scope of the study. The strongest claim is not patient selection, but that a curated kinase-inhibitor subset can test whether molecular context adds externally reproducible information. That claim is supported by GDSC1 validation, bootstrap gain analysis, continuous-response checks, endpoint sensitivity analysis and the ERK/MAPK subgroup signal. At the same time, the weaker RTK and PRISM results show that model transferability remains pathway- and platform-dependent. Because the present study does not include patient-derived xenografts, organoids or patient clinical-response datasets, prospective validation in more clinically representative systems remains necessary before clinical translation.

## 5 Limitations

Several limitations remain. First, the study uses public 2D cell-line screens and does not include patient-level clinical response. Patient-derived xenograft pharmacogenomics illustrates the kind of downstream validation layer that would be needed before making clinical-response claims (Mer et al., 2019). Prospective validation in patient-derived xenografts, organoids or clinical-response datasets remains necessary before clinical translation. Second, binary sensitivity was defined primarily by drug-specific medians. This intra-drug median binarization is useful as a benchmarking endpoint because it balances sensitive and resistant labels within each drug, but it should not be interpreted as a clinical response definition. The endpoint sensitivity analysis showed that predictive signal was retained under IC50 < 1 uM, fixed AUC thresholds and waterfall-derived sensitivity endpoints. However, these labels were still derived from public 2D cell-line screens and should not be interpreted as patient-response criteria. Third, PRISM validation used fewer shared drugs than GDSC validation and changed both assay platform and overlap structure. The PRISM result therefore provides a cross-platform stress test rather than a direct replication of the GDSC validation chain. Fourth, the feature engineering is intentionally conservative. The design does not include morphology, docking, protein-structure simulation or patient-derived omics, because the present study prioritises transparent feature matching and external validation. In this context, RTK signalling represents a limitation of the current interpretable summary-feature approach and a priority area for richer mechanistic modelling. Future work should test the ERK/MAPK signal in patient-derived models or experimental assays and develop richer mechanistic representations for RTK inhibitor response.

## 6 Conclusion

This study supports a focused public-data contribution to kinase-inhibitor response modelling. PKIDB-informed target and pathway summaries, when combined with DepMap molecular profiles, improved externally reproducible response prediction across GDSC releases and showed the clearest signal in ERK/MAPK inhibitors. Cross-platform PRISM validation and RTK subgroup analyses further defined the limits of model portability and pathway-level generalisability. These findings support the value of transparent public-data provenance and tiered validation for pharmacogenomic modelling, while highlighting the need for patient-derived and clinical-response validation before clinical translation.

## Data Availability

The datasets presented in this study can be found in online repositories. The names of the repository/repositories and accession number(s) can be found below: The datasets analyzed in this study are publicly available from PKIDB, GDSC release 8.5, DepMap 26Q1 and the PRISM Repurposing secondary screen. Processed source tables, figure source data and repository links should be verified and added before final submission, or made available by the corresponding author on reasonable request.
