# Supplementary material for: PKIDB-informed molecular profiling improves reproducible prediction of cancer kinase-inhibitor response
Source: Front Pharmacol. 2026 Sep 17;17:1923164. doi: 10.3389/fphar.2026.1923164 (PMC13627788; doi:10.3389/fphar.2026.1923164)
Supplement: Supplementary file 1 [file DataSheet1.docx]

Supplementary material

**Supplementary Figure S1. Endpoint sensitivity analysis under fixed-threshold and waterfall-derived response definitions.** (A) Agreement between alternative sensitivity labels and the original drug-median sensitivity labels. Alternative endpoints included IC50 < 1 µM, LN_IC50 waterfall inflection, AUC < 0.80, AUC < 0.85, AUC < 0.90 and AUC waterfall inflection. (B) External validation performance under alternative endpoint definitions, reported as ROC AUC and average precision (AP). The dashed line indicates random-level ROC AUC. (C) Positive-class fraction under each alternative endpoint definition, showing class-balance shifts relative to the balanced median-split design. The dashed line indicates a 0.50 positive fraction.(D) Distribution of drug-specific waterfall-derived AUC cutoffs. Dashed horizontal lines indicate fixed AUC thresholds of 0.80, 0.85 and 0.90. (E) Distribution of drug-specific waterfall-derived LN_IC50 cutoffs. The dashed horizontal line indicates the IC50 < 1 µM threshold, corresponding to LN_IC50 < 0. (F) Cross-tabulation of median-based and waterfall-derived AUC sensitivity labels. Cell values show the number and percentage of observations in each label combination.

**Supplementary Figure S2. ERK/MAPK and RTK pathway comparison using feature ablation, molecular-summary signal and drug-level heterogeneity.** (A) Feature-set ablation comparing GDSC1 external ROC AUC for ERK/MAPK and RTK signalling models across drug-lineage features, expression summaries, CRISPR summaries, combined expression plus CRISPR summaries and full molecular summaries. (B) Added ROC AUC gain of molecular-summary features over drug-lineage features for ERK/MAPK and RTK signalling. (C) Putative target-count distribution for ERK/MAPK and RTK inhibitors, showing greater target-spectrum complexity in RTK-associated inhibitors. (D) Median absolute Spearman association between selected molecular-summary features and drug response, comparing pathway-level and target-level expression and CRISPR dependency summaries. (E) Pathway-specific model importance grouped by feature category, including lineage, drug/target identity, mutation, copy number, CRISPR dependency and expression. (F) Drug-level GDSC1 external ROC AUC values for representative ERK/MAPK and RTK-associated inhibitors, illustrating stronger and more consistent performance for ERK/MAPK inhibitors and greater heterogeneity among RTK-associated inhibitors.

**Supplementary Figure S3. PRISM performance-drop and cross-platform response-discordance analysis.** (A) Comparison of GDSC1 external validation performance and PRISM cross-platform validation performance, measured by ROC AUC. (B) Median-label concordance matrix for exact shared GDSC1-PRISM drug-model pairs. Cell values show the number and percentage of paired observations classified as sensitive or resistant in each platform. (C) Distribution of GDSC1 AUC and PRISM AUC values in exact shared drug-model pairs, showing a modest response-distribution shift between platforms. (D) Drugs with the highest GDSC1-PRISM median-label discordance fractions. ERK/MAPK-associated drugs are shown in green, RTK-associated drugs in red and other kinase groups in grey. (E) Drug-level dose-range context in GDSC1 and PRISM, summarized as log10 dose-range width. (F) Pathway-level GDSC1-PRISM response concordance, measured by Spearman correlation across exact shared drug-model pairs.
